# Progress in cancer epidemiology: avoided deaths in Europe over the last three decades

**DOI:** 10.1097/CEJ.0000000000000714

**Published:** 2021-08-26

**Authors:** Carlo La Vecchia, Eva Negri, Greta Carioli

**Affiliations:** a Department of Clinical Sciences and Community Health, Università degli Studi di Milano, Milan; b Department of Humanities, Università Telematica Pegaso, Naples; c FROM Research Foundation, Papa Giovanni XXIII Hospital, Bergamo, Italy

**Keywords:** avoided deaths, cancer, Europe, mortality rates, prediction models

## Abstract

Progress in cancer epidemiology and prevention has been a key determinant of the fall in cancer mortality in Europe. Using mortality and population figures from the WHO and Eurostat databases, we estimated the number of averted cancer deaths in the EU27 over the period 1989–2021, for both sexes, for all cancers, and nine major cancer sites. We also computed the avoided deaths for all cancers in five major European countries and the UK. We estimated a total of 4 958 000 (3 339 000 men and 1 619 000 women) avoided deaths for all neoplasms over the period 1989–2021 and 348 000 (246 000 men and 102 000 women) in 2021 alone in the EU27. For both sexes, we estimated 1 679 000 avoided deaths for stomach cancer, 747 000 for colorectum, 227 000 for bladder, 102 000 for leukemias. Avoided deaths for lung cancer accounted for 1 156 000 in men, while no reduction was estimated for women. For breast and uterine cancer, avoided deaths were about 300 000, for ovary 105 000 and for prostate 352 000. In the UK, a total of 1 061 000 (721 000 men and 340 000 women) deaths was avoided. Elimination of tobacco may avoid a further 20% of cancer mortality by 2050. Control of alcohol, overweight and obesity, and occupational and environmental carcinogens may avoid an additional 10% of cancer deaths. A similar reduction may be due to optimal adoption of cervical, colorectal, breast, and probably, lung and prostate cancer screening. Thus, primary and secondary cancer prevention can avoid an additional third of cancer deaths in Europe up to 2050.

## Introduction

Cancer mortality in Europe has been steadily increasing until the late 1980s, but has levelled off and declined over the last three decades (Rosso *et al.*, 2018).

A large portion of such decline is due to progresses in cancer epidemiology and prevention, including mainly, but not only, tobacco control in men.

We estimated, therefore, the number of avoided cancer deaths over the last three decades, and discussed the key role of epidemiology and prevention in these figures.

## Materials and methods

We obtained official death certification data from the WHO database for total cancer mortality and 9 major cancer sites. Cancer deaths were recoded according to the 10th ICD Revision: total cancers (C00–D48), stomach (C16), colorectum (C17–C21, C26), lung (C33–C34), breast (C50), uterus (cervix and corpus) (C53–C55), ovary (C56), prostate (C61), bladder (C67) and leukemias (C91–C95). We obtained resident population estimates from the same WHO database, and when data were missing, from Eurostat database. We derived figures from 1970 to 2015 for the EU (current 27 member states; data were missing for Cyprus) and for its five most populous countries (France, Germany, Italy, Spain and Poland), and the UK.

We calculated country- and sex-specific deaths rates for each 5-year age group (0–4 to 85+ years) and calendar year. To predict figures and rates, we fitted a logarithmic Poisson joinpoint regression model to each 5-year age-specific number of certified deaths, setting a maximum of five joinpoints; we identified the most recent trend segment. We estimated age-specific numbers of deaths and the corresponding 95% prediction intervals (PIs) for 2021 by fitting a linear regression to the mortality data from each age group over the most recent trend segment identified by the joinpoint model. Using the matrices of predicted age-specific numbers of deaths obtained from our model and predicted populations retrieved from Eurostat, we computed predicted age-specific death rates with 95% PIs (Carioli *et al.*, 2021).

We estimated the number of averted cancer deaths for the EU27 for total neoplasms and the nine major cancer sites, for men and women, over the 1989–2021 period, by comparing observed deaths and expected ones on the basis of the 1988 age-specific peak rate. We also estimated the total neoplasms avoided deaths for the five major European countries and for the UK over the 1989–2021 period.

## Results

**Fig. 1:**
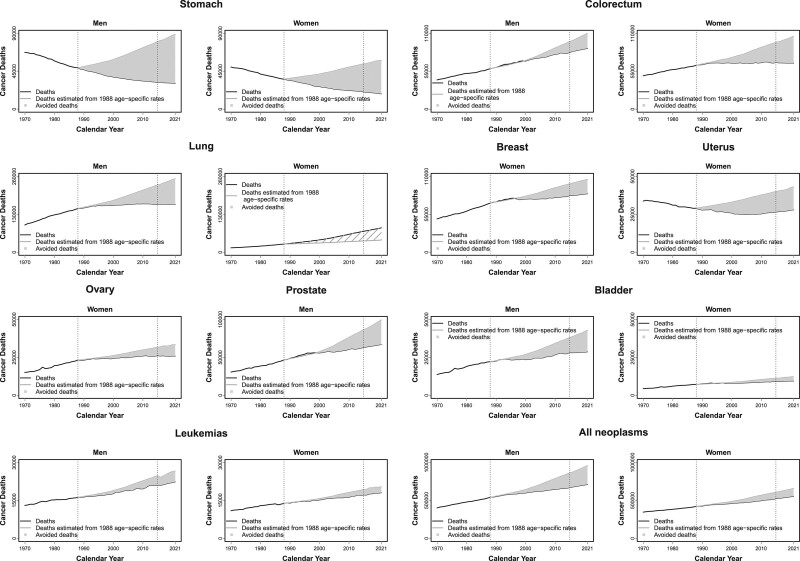
Avoided cancer deaths for all neoplasms and 9 major cancer sites for the EU27 men and women between the top rate in 1988 and 2021 (light grey area); observed numbers of cancer deaths from 1970 to 2015 and predicted cancer deaths from 2016 to 2021 (black line); estimated numbers of cancer deaths by applying 1988 age-specific peak mortality rate (dark grey).

Table 1 and Figure 1 show the estimated number of averted cancer deaths for the EU27 by sex and cancer site from 1989 to 2021, as well as in 2021 alone. In the EU27, we estimated a total of 4 958 000 (3 339 000 men and 1 619 000 women) avoided deaths for all neoplasms over the 33-year period and 348 000 (246 000 men and 102 000 women) in 2021 alone. For both sexes, we estimated 1 679 000 avoided deaths for stomach cancer, 747 000 for colorectum, 227 000 for bladder and 102 000 for leukemias. Avoided deaths for lung cancer accounted for 1 156 000 in men, while a rise of deaths for this cancer was estimated in women, as compared to the 1988 rates, 622 000 deaths over the entire period considered and 42 000 in 2021. For breast and uterine cancer, avoided deaths since 1989 were about 300 000, for ovary 105 000 and for prostate 352 000.

**Table 1:** Avoided cancer deaths for total neoplasms and for nine major cancer sites in men and women for the EU27 as a whole, over the period 1989–2021 and in 2021 alone

	Avoided deaths over 1989–2021		Avoided deaths in 2021	
	Men	Women	Men	Women
Stomach	987 000	692 000	58 000	39 000
Colorectum	218 000	529 000	22 000	39 000
Lung	1 156 000	-	87 000	-
Breast	-	301 000	-	21 000
Uterus (Cervix & Corpus)	-	301 000	-	15 000
Ovary	-	105 000	-	7000
Prostate	352 000	-	32 000	-
Bladder	190 000	37 000	14 000	3000
Leukemias	65 000	37 000	4000	2000
All neoplasms	3 339 000	1 619 000	246 000	102 000

**Fig. 2:**
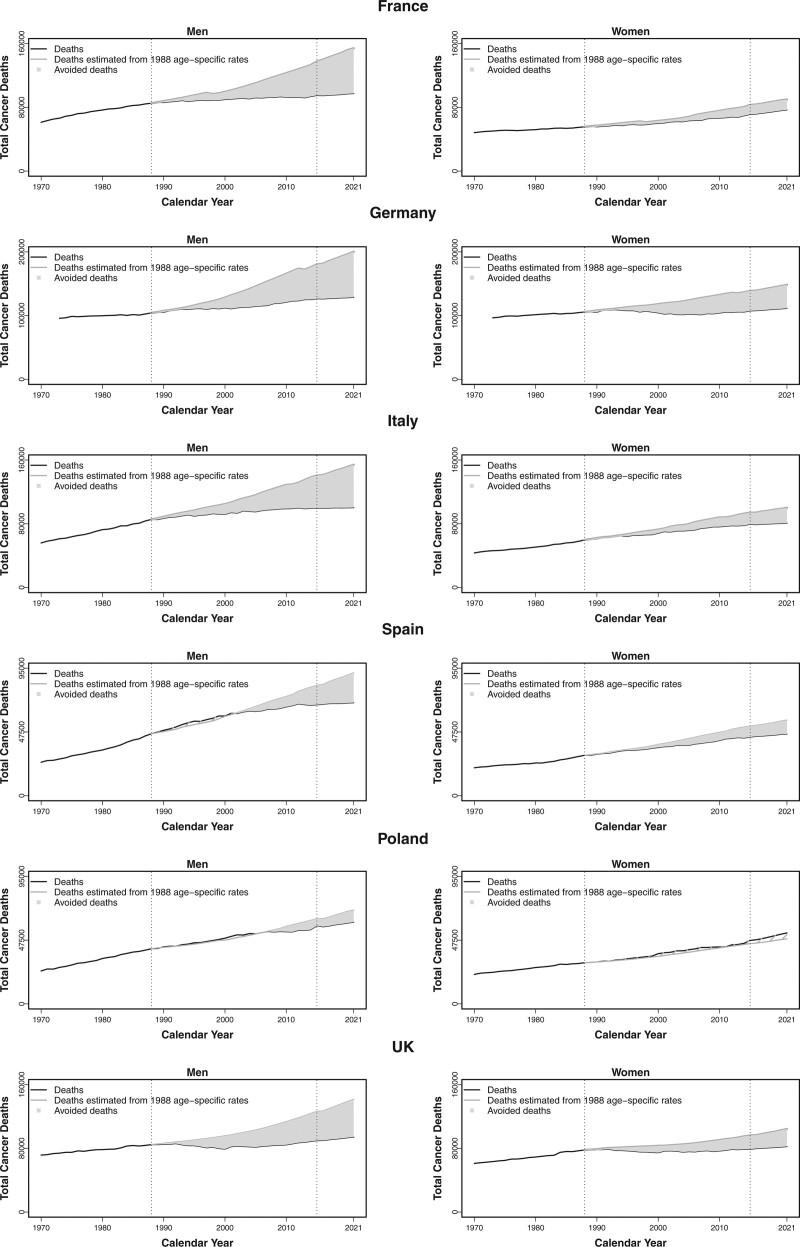
Avoided cancer deaths for all neoplasms, in men and women, in the 5 major European countries and the UK between the top rate in 1988 and 2021 (light grey area); observed numbers of total cancer deaths from 1970 to 2015 and predicted cancer deaths from 2016 to 2021 (black line); estimated numbers of total cancer deaths by applying 1988 age-specific peak mortality rate (dark grey).

Table 2 and Figure 2 show the estimated number of averted deaths for all neoplasms for the five major European countries and the UK, by sex, from 1989 to 2021, as well as in 2021 alone. Over the 33-year period, in France, a total of 972 000 cancer deaths were avoided (759 000 in men and 213 000 in women), in Germany 1 670 000 (1 026 000 in men and 644 000 in women), in Italy 1 025 000 (748 000 in men and 277 000 in women), in Spain 334 000 (187 000 in men and 147 000 in women), in the UK 1 061 000 (721 000 in men and 340 000 in women). In Poland, 70 000 deaths were averted for men, while no reduction was estimated for women.

**Table 2:** Avoided cancer deaths for total neoplasms in men and women for the five major European countries and the UK, over the period 1989–2021 and 2021 alone

	Avoided deaths over 1989–2021		Avoided deaths in 2021	
	Men	Women	Men	Women
France	759 000	213 000	57 000	13 000
Germany	1 026 000	644 000	71 000	37 000
Italy	748 000	277 000	54 000	19 000
Spain	187 000	147 000	22 000	10 000
Poland	70 000	-	9000	-
UK	721 000	340 000	47 000	22 000

## Discussion

Primary and secondary prevention had a key role in the substantial fall in cancer mortality – around five million – over the last three decades in Europe.

At least 80% of the decline in lung cancer mortality in men is due to reduced smoking prevalence (Malhotra *et al.*, 2016). Since in several European countries the decline in smoking prevalence has been levelling off over the last few years, additional intervention on tobacco control in European men is required. Tobacco control in women remains a key priority since female lung cancer rates are still rising. The reduced smoking prevalence in men had a favourable impact on the prevention and hence reduced mortality of other tobacco-related cancers, including bladder, stomach and colorectum.

The impact of alcohol, the second major cause of cancer in Europe, on cancer trends is heterogeneous, since alcohol consumption has substantially declined in southern Europe over the last few decades, but has increased in most central and northern Europe (La Vecchia *et al.*, 2014).

Primary prevention had also a major role on the reduction of gastric cancer mortality, especially through eradication of *Helicobacter pylori*, but also through improvement in food conservation, diet and nutrition and tobacco control (Lyons *et al.*, 2019).

Another aspect of primary prevention is vaccination against hepatitis B on liver cancer (available across Europe in the late 1980s) (Bosetti *et al.*, 2014) and HPV on cervical, oropharyngeal and anal cancers (available around 2005) (Villain *et al.*, 2015). The impact of these interventions (particularly that of HPV) on cancer mortality is however still moderate but will increase in the future.

The avoidance of cervical cancer deaths is due to secondary prevention, mainly PAP test and, more recently HPV testing. There is however scope for improving organised cervical cancer screening programs, particularly in central and eastern Europe, where rates remain unacceptably high (Wojtyla *et al.*, 2020).

Secondary prevention had a relevant role in the avoidance of colorectal and breast cancer deaths. Various approaches to screening and early diagnosis of colorectal cancer, including faecal occult blood testing, colonoscopy and sigmoidoscopy can reduce incidence (by eliminating pre-neoplastic lesions) and mortality of colorectal cancer by 30–40%. A similar estimate can be applied to mammography in the avoidance of breast cancer mortality, which may account for about a third of the substantial reduction in breast cancer mortality observed in Europe. Improved treatment is responsible for an even larger proportion in breast cancer deaths (Carioli *et al.*, 2017; Tabár *et al.*, 2019). Part of the fall in prostate cancer mortality is likely due to screening and early diagnosis through PSA (Cuzick *et al.*, 2014), though its impact remains difficult to quantify. Low-dose computed tomography may reduce lung cancer mortality (Rota *et al.*, 2019), but its adoption is still limited across Europe. The Covid pandemic may have an unfavourable impact on cancer screening, if the organised programs are not rapidly re-installed (Carioli *et al.*, 2021; Martin-Moreno and Lessof, 2021)

## Conclusion

Cancer prevention remains a key factor in cancer control in Europe. Elimination of tobacco may avoid a further 20% of cancer mortality by 2050. Reduction of alcohol drinking, control of overweight and obesity, and further improvements in exposures to occupational and environmental carcinogens may avoid an additional 10% of cancers and cancer deaths. Widespread adoption of cervical, colorectal, breast and probably lung and prostate cancer screening may contribute to a further reduction by 5–10% in cancer mortality. Thus, primary and secondary cancer prevention can in principle additionally avoid over a third of cancer deaths in Europe by 2050.

## Acknowledgements

This work was conducted with the contribution of the Italian Association for Cancer Research (AIRC, project N. 22987).

### Conflicts of interest

There are no conflicts of interest.

## References

[R1] Bosetti C Turati F La Vecchia C (2014). Hepatocellular carcinoma epidemiology. Best Pract Res Clin Gastroenterol 28:753–770. 25260306 10.1016/j.bpg.2014.08.007

[R2] Carioli G Malvezzi M Rodriguez T Bertuccio P Negri E La Vecchia C (2017). Trends and predictions to 2020 in breast cancer mortality in Europe. Breast 36:89–95. 28988610 10.1016/j.breast.2017.06.003

[R3] Carioli G Malvezzi M Bertuccio P Boffetta P Levi F La Vecchia C Negri E (2021). European cancer mortality predictions for the year 2021 with focus on pancreatic and female lung cancer. Ann Oncol 32:478–487. 33626377 10.1016/j.annonc.2021.01.006

[R4] Cuzick J Thorat MA Andriole G Brawley OW Brown PH Culig Z. (2014). Prevention and early detection of prostate cancer. Lancet Oncol 15:e484–e492. 25281467 10.1016/S1470-2045(14)70211-6 PMC4203149

[R5] La Vecchia C Bosetti C Bertuccio P Castro C Pelucchi C Negri E (2014). Trends in alcohol consumption in Europe and their impact on major alcohol-related cancers. Eur J Cancer Prev 23:319–322. 24045697 10.1097/CEJ.0b013e32836562f1

[R6] Lyons K Le LC Pham YT Borron C Park JY Tran CTD. (2019). Gastric cancer: epidemiology, biology, and prevention: a mini review. Eur J Cancer Prev 28:397–412. 31386635 10.1097/CEJ.0000000000000480

[R7] Malhotra J Malvezzi M Negri E La Vecchia C Boffetta P (2016). Risk factors for lung cancer worldwide. Eur Respir J 48:889–902. 27174888 10.1183/13993003.00359-2016

[R8] Martin-Moreno JM Lessof S (2021). Predictions of cancer mortality in Europe in 2021: room for hope in the shadow of COVID-19? Ann Oncol 32:425–426. 33626376 10.1016/j.annonc.2021.02.001 PMC8970709

[R9] Rosso T Malvezzi M Bosetti C Bertuccio P Negri E La Vecchia C (2018). Cancer mortality in Europe, 1970-2009: an age, period, and cohort analysis. Eur J Cancer Prev 27:88–102. 27472086 10.1097/CEJ.0000000000000282

[R10] Rota M Pizzato M La Vecchia C Boffetta P (2019). Efficacy of lung cancer screening appears to increase with prolonged intervention: results from the MILD trial and a meta-analysis. Ann Oncol 30:1040–1043. 31046087 10.1093/annonc/mdz145

[R11] Tabár L Dean PB Chen TH Yen AM Chen SL Fann JC. (2019). The incidence of fatal breast cancer measures the increased effectiveness of therapy in women participating in mammography screening. Cancer 125:515–523. 30411328 10.1002/cncr.31840 PMC6588008

[R12] Villain P Gonzalez P Almonte M Franceschi S Dillner J Anttila A. (2015). European code against cancer 4^th^ edition: infections and cancer. Cancer Epidemiol 39 (Suppl 1):S120–S138. 26589774 10.1016/j.canep.2015.10.006

[R13] Wojtyla C Janik-Koncewicz K La Vecchia C (2020). Cervical cancer mortality in young adult European women. Eur J Cancer 126:56–64. 31918234 10.1016/j.ejca.2019.11.018

